# Atypical cause of radiating leg pain: Visceral referred pain due to ulcerative colitis

**DOI:** 10.1016/j.radcr.2024.09.109

**Published:** 2024-10-14

**Authors:** Rani De Pauw, Daute De Brucker, Annick Viaene, Luc Vanden Bossche, Bartel Thomas

**Affiliations:** a University Hospital Ghent, Departement Physical and Rehabilitation Medicine, Belgium; b General City Hospital Aalst, Departement Physical and Rehabilitation Medicine, Belgium; c University Hospital Ghent, Departement Radiology, Belgium

**Keywords:** Neuropathic pain, Sciatica, Ulcerative colitis, Visceral reference

## Abstract

Radiating leg pain can arise from a variety of etiologies in clinical practice, the most prevalent being neuropathic or musculoskeletal in origin. In rare cases an intra-abdominal pathology may also be the underlying cause. A case is reported of a 30-year old female patient who presented with neuropathic pain in the left groin region, radiating to the left thigh since four months. The pain was ultimately attributed to visceral referred pain associated with underlying ulcerative colitis (UC). The pathophysiological mechanism behind visceral referred pain originating from the distal colon —a region potentially affected in inflammatory bowel diseases such as UC— is likely due to the convergent input of somatic and visceral (lumbar splanchnic) afferent fibers at the L1-L2 level of the lumbar spinal cord, causing referred pain to the L1 and L2 dermatomes, corresponding to the groin and thigh. In this case report, we aim to underline the possibility of referred visceral pain as an uncommon etiology in unexplained radiating leg pain and the importance of thorough medical history and diagnostic evaluation.

## Introduction

Radiating leg pain is a common complaint in both general and specialized medical practice with a broad differential diagnosis. Uncommonly, an inflammatory intra-abdominal condition such as ulcerative colitis (UC) can present as sciatica through visceral referred pain. UC is a chronic, immune-mediated inflammatory disease of unknown etiology that primarily affects the mucosa and submucosa of the rectum with potential extension into the colon. While UC can occur at any age, it usually develops between the ages of 15 and 30 years and has a rising incidence with nearly one million affected individuals in both North America and Europe. The incidence in other regions of the world remains unclear [1,2].

## Case presentation

A 30-year-old European woman was referred to the Physical Medicine and Rehabilitation Department with neuropathic pain in the left groin radiating to the left thigh and associated subjective weakness for approximately four months. Despite a healthy and active lifestyle, having completed a marathon six months prior, she now struggled to walk, run, or cycle due to the pain which also persisted at night. The pain was not exercise-related and medications such as Amitriptyline and Tramadol provided minimal relief. She had no other neurological or systemic symptoms and no cardiovascular risk factors.

On physical examination, deep palpation of the left iliac fossa elicited tenderness and palpation of the posterior superior iliac crest was painful. Hypoesthesia of the left thigh was noted during fine touch testing, although it did not correspond to a clearly defined dermatome. Further examination of the lumbar spine, left hip, knee, and abdominal muscles was normal, as did the neurological examination and assessment of peripheral arterial pulses. Previous investigations by the Orthopedic and Neurology Departments included an ultrasound of the groin and hip, X-rays of the hip and pelvis, MRI scans of the hip, complete spine, and brain, as well as EMG and SSEP studies of the lower limbs, all of which yielded normal results. Additional investigations including CT angiography of the lower limbs, extensive blood tests (including viral serology, auto-immune antibodies, protein electrophoresis, sedimentation rate, CRP, and CK levels), and a gynecological examination also showed no abnormalities. Given the broad differential diagnosis of radiating leg pain and the initial exclusion of (1) locomotor causes, including hip pathology or tendinopathy (gluteus, rectus femoris, psoas), (2) lumbosacral pathology, including mechanical (degenerative joint disease, facet syndrome, discopathy) or inflammatory (sacroiliitis, ankylosing spondylitis) conditions, and (3) neuropathic disorders, such as plexus disorders, radiculopathy, or peripheral neuropathy, the patient was admitted to the Radiology Department for further evaluation of suspected pelvic pathology. This included consideration of osteitis pubis, obturator hernia or visceral irritation due to intra-abdominal (gastrointestinal, urological, or gynecological) inflammation or infection (including a psoas abscess).

**Fig. 1:**
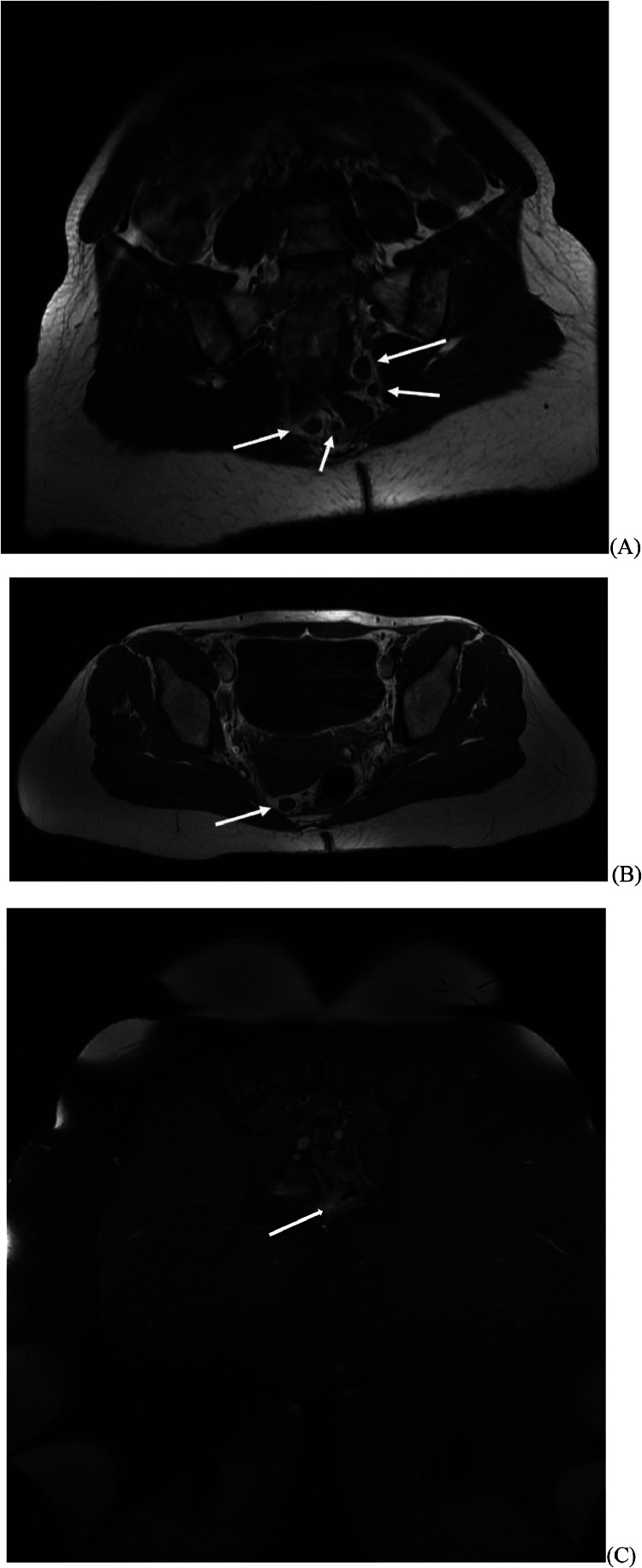
White arrows on axial T1 (fast spin echo) image showing adenopathies surrounding the rectum/rectosigmoid junction (A, B), on coronal PD fat-saturated image displaying edematous wall thickening of the rectum (C).

**Fig. 2:**
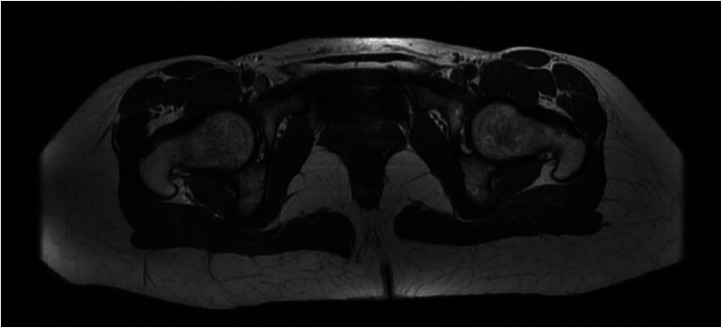
Axial T1 (fast spin echo) image showing an intact pelvic floor and no herniation in the obturator foramen.

A pelvic MRI requested by our department (Fig. 1) revealed perirectal adenopathy and focal rectal wall thickening at the rectosigmoid junction. No obturator hernia could be found (Fig. 2). The patient was immediately referred to Gastroenterology Department for a left-sided colonoscopy which revealed an inflammatory, granular appearance extending from 0 to 10 cm from the anal margin accompanied by mucus formation. Histopathological examination of three mucosal biopsies demonstrated colonic mucosa with basal lymphoplasmacytosis and a dense chronic inflammatory infiltrate with mild activity, findings consistent with inflammatory bowel disease. Upon further inquiry, the patient reported a long-standing history of altered bowel habits with occasional blood in the stool. An additional total colonoscopy confirmed proctitis (Mayo score 2) with histopathological analysis of multiple biopsies revealing normal ileal and colonic tissue, but clear evidence of chronic colitis with severe activity in all rectal biopsies.

The patient was treated by the Gastroenterology Department with Mesalazine (5-aminosalicylic acid) suppositories 1 g/day for 14 days, which partially ameliorated her radiating leg pain. Oral Beclomethasone 5mg/day resulted in the complete disappearance of symptoms after ten days. Continuing this treatment for 14 days led to mucosal improvement, as seen in a follow-up colonoscopy. Oral Beclomethasone 5 mg/day was continued for another six weeks, alternating with Mesalazine suppositories 1 g/day or Beclomethasone suppositories 6 mg/day. A second follow-up colonoscopy after six weeks showed complete remission of UC with a normal endoscopic appearance and the patient remains symptom-free upon today.

## Discussion

The initial presentation of UC typically includes symptoms of rectal inflammation such as bleeding, urgency, diarrhea, and a sensation of incomplete evacuation [2]. However, extra-intestinal manifestations (EIM) can also occur. These EIMs may coincide with the diagnosis of inflammatory bowel disease (IBD) or precede it. According to the European Crohn's and Colitis Organisation (ECCO) up to 50% of patients with IBD have at least one EIM, which can affect any system —with musculoskeletal complaints being the most common— and increase morbidity [3]. Some EIMs, such as primary sclerosing cholangitis or thromboembolic events, can even lead to increased mortality. Despite the overall decrease in colorectal carcinoma prevalence among IBD patients due to improved therapies, these patients are still twice as likely to develop dysplasia and colorectal cancer compared to the general population. The overall risk of colorectal carcinoma is estimated to be between 1.1% and 5.4% over 20 years influenced by factors such as gender, early-age diagnosis, extensive inflammatory involvement, untreated dysplasia, the development of strictures, and the presence of primary sclerosing cholangitis. When UC is confined to the rectum, there is no increased risk of colorectal malignancy [4].

Given the significant improvement in symptoms following the appropriate treatment of inflammatory proctitis, as well as endoscopic remission of UC, the radiating neuropathic pain in the patient's left leg is attributed to visceral referred pain caused by active inflammation of the distal colon and rectum due to UC.

Despite extensive research, the complete mechanism of visceral pain remains inadequately understood due to its diverse manifestations and the challenges of studying it in animal models. It is well established that visceral referred pain arises from hypersensitization of visceral afferent fibers, which converge with somatic afferent fibers at the central nervous system (CNS) level, leading to dysregulation of efferent spinal nociceptive pathways [5]. The descending colon and rectum, primarily affected in UC, are innervated by the lumbar splanchnic nerves [6], which originate from branches of the L1-L2 sympathetic nervous system [7]. Chronic inflammation of the intestinal mucosa in UC results in hyperexcitability of visceral nociceptive neurons in the dorsal horn of the spinal cord at the L1-L2 level, mediated by stretch receptors in the gut [2]. This increased visceral input subsequently leads to heightened nociceptive traffic to the CNS [8,9]. Additionally, the stimulated sensory neurons within the CNS receive convergent input from other somatic afferent fibers, such as those from the skin, via the dorsal horn. This convergence can result in secondary hypersensitivity in the referred area or dermatome [9], in this case, the thigh or upper leg (L1-L2) [10]. More specifically, afferent signals originating from visceral organs project to the dorsal horn of the spinal cord, where they activate three distinct postsynaptic pathways responsible for spinal and supraspinal autonomic reflexes via higher centers located in the amygdala and hypothalamus, which are involved in cognitive evaluation. Consequently, patients with inflammatory bowel disease (IBD) also exhibit increased activation of brain regions associated with pain modulation and emotional arousal. Prolonged hyperexcitability of visceral afferents in UC may thus lead to chronic sensitization of these brain regions associated with pain modulation, resulting in chronic visceral referred pain [11].

Radiating leg pain in UC may also be due EIMs affecting the musculoskeletal system. EIMs are categorized based on their underlying cause, which may include distant inflammation, treatment effects, systemic inflammation, or associations with other immune-mediated conditions. Axial and nonaxial spondyloarthropathies, classic EIMs resulting from distant inflammation [3], can also produce pseudoradicular pain that radiates to the lower limb.

In conclusion, visceral referred pain from intra-abdominal or pelvic pathology should be considered in patients with radiating leg pain from unknown etiology. This case report involved a case concerning ulcerative colitis (UC) causing radiating leg pain in a 30-years old woman. A colonoscopy should be performed to reveal active inflammation of the rectum and/or colon. These patients show significant improvement with the correct treatment of the underlying inflammatory proctitis and/or colitis.

## Patient consent

Complete written informed consent was obtained from the patient for the publication of this study and accompanying images.
